# Visualisation to enhance biomechanical tuning of ankle-foot orthoses (AFOs) in stroke: study protocol for a randomised controlled trial

**DOI:** 10.1186/1745-6215-12-254

**Published:** 2011-12-05

**Authors:** Bruce Carse, Roy J Bowers, Barry C Meadows, Philip J Rowe

**Affiliations:** 1 Bioengineering Department, University of Strathclyde, Glasgow, UK; 2 National Centre for Prosthetics and Orthotics, University of Strathclyde, Glasgow, UK; 3 WestMARC, Southern General Hospital, Glasgow, UK

**Keywords:** Ankle-foot orthosis, biomechanics, gait analysis, visualisation, stroke, hemiplegia, 3D motion analysis

## Abstract

**Background:**

There are a number of gaps in the evidence base for the use of ankle-foot orthoses for stroke patients. Three dimensional motion analysis offers an ideal method for objectively obtaining biomechanical gait data from stroke patients, however there are a number of major barriers to its use in routine clinical practice. One significant problem is the way in which the biomechanical data generated by these systems is presented. Through the careful design of bespoke biomechanical visualisation software it may be possible to present such data in novel ways to improve clinical decision making, track progress and increase patient understanding in the context of ankle-foot orthosis tuning.

**Methods:**

A single-blind randomised controlled trial will be used to compare the use of biomechanical visualisation software in ankle-foot orthosis tuning against standard care (tuning using observation alone). Participants (n = 70) will have experienced a recent hemiplegia (1-12 months) and will be identified by their care team as being suitable candidates for a rigid ankle-foot orthosis. The primary outcome measure will be walking velocity. Secondary outcome measures include; lower limb joint kinematics (thigh and shank global orientations) & kinetics (knee and hip flexion/extension moments, ground reaction force FZ_2 _peak magnitude), step length, symmetry ratio based on step length, Modified Ashworth Scale, Modified Rivermead Mobility Index and EuroQol (EQ-5D). Additional qualitative measures will also be taken from participants (patients and clinicians) at the beginning and end of their participation in the study. The main aim of the study is to determine whether or not the visualisation of biomechanical data can be used to improve the outcomes of tuning ankle-foot orthoses for stroke patients.

**Discussion:**

In addition to answering the primary research question the broad range of measures that will be taken during this study are likely to contribute to a wider understanding of the impact of ankle-foot orthoses on the lives of stroke patients.

**Trial registration number:**

ISRCTN: ISRCTN52126764

## Background

### Post-stroke AFO use

There is currently a limited amount of published evidence that a rigid ankle-foot orthosis (AFO) fitting can improve mobility and indirectly quality of life for stroke patients [1-3]. While assessment for AFO in stroke patients is recommended, the grade of recommendation in recently published Scottish Intercollegiate Guidelines Network (SIGN) clinical guidelines remains low [4]. This low grading of recommendation reflects the lack of evidence on the impact of AFO on both functional and long-term outcomes. It was also noted that there was insufficient evidence regarding the comparative effects of different types of AFO. Guidance on screening and assessment for the provision of AFO for stroke patients is now available in an NHS Scotland Best Practice Statement [5].

### AFO tuning

AFO tuning involves making small dimensional changes at the foot and ankle that can have a significant positive biomechanical effect at the knee and hip in children with cerebral palsy [6-9]. Appendix 7 of the NHS BPS [5] provides guidance on how to tune an AFO effectively for stroke patients. Given the supposed important role of AFO tuning, it is surprising that in previous AFO stroke studies there are limited descriptions of the AFOs used in terms of; position of leg during casting or scanning, materials used, tuning process followed (if any) and heel wedge sizes.

While the majority of AFO tuning studies have considered children with cerebral palsy, one describes a case study with a stroke patient [10]. The authors found that AFO tuning did have a positive effect on stride length and a reduction in knee hyperextension.

### Gait analysis

Observational gait analysis has been shown to be ineffective and unreliable [11,12] while computerised 3D gait analysis results have been shown to have a significant impact on clinical decision making [13,14], thus strengthening the argument for the use of objective computerised gait analysis techniques. Despite computerised gait analysis being shown to have a significant impact on surgical decision making there are a number of perceived barriers to its use. Baker described many of these barriers and identified the interpretation of clinical gait data as being a significant problem [15]. Furthermore, Coutts stated that "interpretation of biomechanical data is complex, time consuming and not readily understood by most therapists" [16]. Other barriers include the cost of the motion analysis equipment, the high level of technical expertise required to operate it and the time needed to collect patient data. Physiotherapists and orthotists in the UK rarely have access to computerised 3D gait laboratories to assist with their clinical decision making [17].

Physiotherapists and orthotists play pivotal roles in the multi-disciplinary team (MDT) approach advocated by the NHS BPS [5] and in the context of AFO tuning there is a clear need for a method of making biomechanical gait data easier to understand through having a user-friendly interface and a rapid presentation of key gait parameters. One study found that there were significant inter-disciplinary differences in how gait data is analysed [17], so there is a need for a common gait analysis language that can be understood by a range of healthcare professionals.

### 3D motion analysis and AFO tuning

The results of a pilot study where a rigorous biomechanical approach to AFO fitting and tuning was used, with ten stroke patients, showed that correct use of AFOs improved a number of important gait characteristics [18]. The results showed significant improvements in ground reaction force (GRF) vector alignment at the hip and an increase in step length for the affected limb as well as an overall increase in walking velocity. Other improvements were also found in GRF alignment at the knee, step length and knee extension of the unaffected limb. Given the small sample sizes used in the pilot study, the more powerful study described here is justified.

Previous work in the area of visualising biomechanical data has shown that animation techniques can enhance both older adult, health professional and design professionals' understanding of the biomechanics of everyday tasks [19]. Given the complex biomechanical nature of AFO tuning, it follows that this activity could benefit from the use of 3D motion analysis combined with a biomechanical visualisation tool (to interpret the data). This may help the MDT administering the treatment package and subsequently improve patient outcomes.

This study constitutes one part of a wider project called **en**vis**age**. The **en**vis**age **project will investigate the use of visualisation software to help communicate biomechanical data in a variety of rehabilitation settings. The project represents a multidisciplinary collaboration between the University of Strathclyde, The Glasgow School of Art and Glasgow Caledonian University.

### Primary research question

Does visualisation of biomechanical data improve the outcomes of tuning ankle-foot orthoses (AFOs) for stroke patients?

### Secondary research questions

1. Can biomechanical visualisation enhance orthotists' and physiotherapists' understanding of biomechanics?

2. Could a visualisation tool facilitate better communication between the members of the multidisciplinary team?

3. Does a patient's understanding of their own biomechanics and corresponding treatment programme change when a visualisation tool is used?

4. Can a database of anonymous gait data on the effects of AFO on stroke patients be created?

## Methods/design

### Study design - Single-blind RCT

The null hypothesis of the study is that there is no difference in outcome measures for stroke patients between observation-based AFO tuning and objective AFO tuning with 3D motion analysis and biomechanical visualisation software. The alternative hypothesis is that objective AFO tuning with biomechanical visualisation software improves outcome measures for stroke patients.

**Figure 1:**
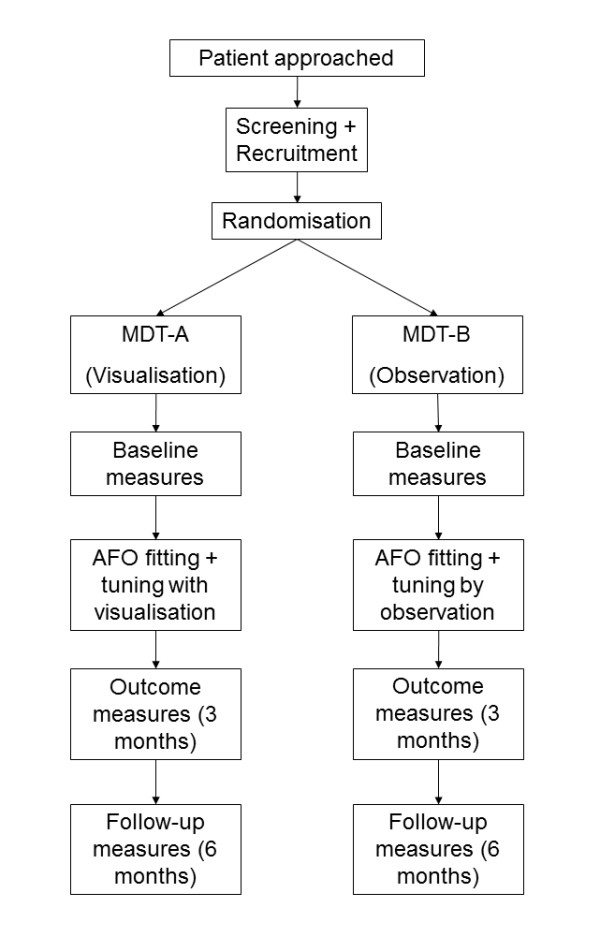
**Flow diagram of trial design showing intervention (visualisation) and non-intervention (observation) arms**.

A single-blind randomised controlled trial (RCT) methodology has been selected to test this hypothesis as shown in Figure 1. The patients will be blinded as to the intervention they will be given, however the clinicians involved will not be blinded. The independent assessor conducting the statistical analysis on the main trial outcome measures will also be blinded. The main benefit of using an RCT is that any potential selection bias and confounding factors will be minimised.

The randomisation sequence will be generated using an independently verified S-PLUS program, by the method of randomised permuted blocks of length four, with participants allocated to interventions in a 1:1 ratio. Researchers will access the random allocations via the study web portal (designed and maintained by The Robertson Centre for Biostatistics at The University of Glasgow) and the sequence will remain concealed until the participant's details are registered on the system.

As patients are recruited to the trial they will be randomly allocated to one of two multidisciplinary teams (MDTs). Each of these MDTs will be made up of a physiotherapist, orthotist and bioengineer. MDT-A will administer the intervention arm of the study, MDT-B will administer the non-intervention arm. In the intervention arm patients will be shown visualisations of their gait patterns and progress throughout the trial. Additionally, the clinicians in MDT-A will also be able to use the gait data and visualisation software to aid their clinical decision making during the AFO tuning session. MDT-B will conduct AFO tuning using standard observational techniques.

Baseline, outcome (3 months) and follow-up (6 months) measurement sessions will be collected by the bioengineer and a third physiotherapist who is independent of the MDTs.

All clinical decision making will be carried out by the physiotherapists and orthotists, while the bioengineer will play a purely technical role collecting data and presenting it to the clinicians and the patient when it is required. The same bioengineer will be used in both MDTs and will be present during all sessions.

Ideally both MDTs would be identical, but this is clearly not possible. Two different MDTs are needed to avoid learning effects in the intervention arm of the study crossing over to the non-intervention and confounding results. In order to balance the MDTs, team members will be selected such that they have similar levels of experience and they will all be required to complete a small amount of basic biomechanics refresher training before the trial commences.

In order to ensure that patients in both arms of the study receive similar AFOs before the tuning stage, a set of design criteria were agreed which both orthotists will follow. These criteria specify the type and thickness of material to be used, angle of ankle dorsiflexion, the pre-tuning shank-to-vertical angle of the AFO when combined with footwear, the trim lines, carbon fibre reinforcements to be used and the positioning/functionality of the straps. See sub-section 'Rigid AFO Design Criteria' for more detail.

### Measurement sessions

#### AFO scanning and fitting

When a patient is recruited to the study they will be randomised and assigned to one arm of the study. The appropriate orthotist will scan them for a rigid AFO and deliver the AFO according to the set of design criteria unique to this study. After scanning and prior to AFO fitting and tuning, the baseline measures can be taken.

#### Baseline

All patients will have baseline gait measurements taken using a 3D motion analysis system. After measurements have been taken, patients in the intervention arm will additionally be shown relevant visualisations which will help to explain and quantify their gait problems.

#### Tuning

Patients in the intervention arm will be given AFO tuning with visualisation. Patients in the non-intervention arm will be given AFO tuning using observational techniques. All patients will have gait measures taken at the end of the tuning process, wearing their tuned AFO.

#### Outcome measures (3 months)

All patients will have 3 month gait measurements taken using a 3D motion analysis system. Patients in the intervention arm will have their progress discussed, aided by visualisations of the data measurements just taken.

#### Follow-up measures (6 months)

All patients will have 6 month gait measurements taken using a 3D motion analysis system. Patients in the intervention arm will have their progress discussed, aided by visualisations of the data measurements just taken.

### Participant recruitment (inclusion/exclusion criteria)

The stroke physiotherapy team will identify stroke patients who are suitable for AFO treatment and will notify the researcher of those considered to meet all of the inclusion/exclusion criteria (see Table 1). The researcher will then inform them of the study and if they are interested they will be given a copy of the 'information for participants' sheet to read. The patient will be given a suitable amount of time (minimum of 48 hours) to consider volunteering. During this time they will be free to ask for more information from the researcher about the study and to consult with friends, carers or relatives.

**Table 1:** Participant inclusion and exclusion criteria

Inclusion criteria	Exclusion criteria
Inpatients who have suffered a recent (1-12 months) hemiplegia	Are unable to give informed consent
Aged 16-80 years	Are unable to walk, even when assisted
Male or Female	Suffer from significant peripheral vascular disease ? not suitable for fitting of AFO
Have difficulty walking, but able to walk with/without assistance	Have any other significant medical problems likely to preclude use of AFO or follow-up
Have difficulty flexing knee and extending hip during walking	
Meet the criteria for AFO referral as outlined in AFO screening tool (NHS BPS, Appendix 9)	
Able to give informed consent	
Able to attend for follow-up at 3 and 6 months	

Communication problems are common in stroke patients, so the patient's care team will be asked to assess the patient's capacity for making informed consent. In addition to providing the 'participant information' sheet the care team will also take the time to verbally explain the trial to the patient if required. It will be made clear to the patient that treatment will not be withheld if they decline to participate in the trial. They will still receive an AFO and an AFO tuning session if they do not volunteer. If they agree to participate in the trial they will be asked to sign a 'research participant consent form'. Should the patient be unable to sign or to mark a document so as to indicate his/her consent, it can be given orally in the presence of at least one witness and recorded in writing. Suitable verbal and graphical explanations will supplement the consent process as necessary on an individual basis. The 'information for participants' sheet makes it clear that the patient is free to withdraw from the study at any time, without giving reason and that withdrawal will not their standard of care nor their future treatment.

### Sample size (power calculation used)

The sample size was decided upon by using data from previous similar studies. A clinically relevant change in walking velocity has been identified as 0.2 m/s [20]. Using conservative figures from a previous RCT [21] the standard deviation of the treatment effect in a control population was 0.24 m/s. This provided a standardised difference statistic of 0.831, which suggests that a sample size of 62 (31 per group) will give 90% power at 5% significance level. Seventy participants (35 per group) will allow for a withdrawal rate of > 10%.

### Location

Participants will be recruited from the stroke wards at the Southern General Hospital, Glasgow, UK. Baseline, tuning, 3 month and 6 month sessions will all take place at West of Scotland Mobility and Rehabilitation Centre (WestMARC) at the Southern General Hospital, Glasgow, UK.

### Data collection equipment

3D motion analysis will be carried out using and 8-camera Vicon 612 motion analysis system (Oxford Metrics, UK) in conjunction with 2 AMTI BP400600 force platforms.

### Quantitative data collected (Primary and Secondary outcome measures)

The primary outcome measure for this study is walking velocity [22]. Secondary outcome measures include; lower limb joint kinematics (thigh and shank global orientations) & kinetics (knee and hip flexion/extension moments, ground reaction force FZ_2 _peak magnitude), step length, symmetry ratio based on step length [23], Modified Ashworth Scale [24], Modified Rivermead Mobility Index [25,26], EuroQol (EQ-5D) [27].

### Qualitative data

The quantitative outcome measures of the RCT are not sufficient to answer all of the study's secondary research questions, so through the use of qualitative research methods it is anticipated that an enhanced understanding of the effects of using visualisations in AFO tuning can be acquired [28]. This part of the study will explore what effect the use of the visualisation tool has on the experiences and understanding of patients and clinicians involved in the study, as well as what effect it has on the patient-clinician interactions.

One-to-one semi-structured interviews will be conducted with all clinicians and patients both before and after their participation in the study.

Before the study, after they have consented to participate, all patients will be required to complete short (15 minute) interviews. The main focus of the questions will be on their expectations of the AFO process, their personal goals and their personal perception of their walking ability at the baseline stage. Clinicians will also complete slightly longer (30 minute) interviews which concentrate on how confident they feel about biomechanics, what they look for during observational gait analysis (are there any inter-disciplinary differences?), attitudes towards 3D motion analysis and perceived difficulties in communicating gait abnormalities to health professionals from other disciplines.

After their participation in the study all patients will complete 30 minute interviews, covering a range of topics including; patient-clinician communication, understanding of their treatment, motivation levels, personal goals achieved, impact of visualisations. Their personal perception of their walking ability will also be reassessed. Clinicians will also complete 45 minute interviews, which will investigate themes such as; evidence of enhanced understanding of biomechanics, team working, perceived benefits/drawbacks of using software and confidence in their clinical decision making.

### Statistical Analysis

Quantitative data from the four patient measurement sessions (baseline gait, AFO tuning, three and six month gait) will be analysed on an 'intention to treat' basis. Data will be held independently on a bespoke database at the Robertson Centre for Biostatistics at the University of Glasgow, and independent assessors comparing data sets will be blinded as to which arm of the trial each patient was allocated to.

The primary emphasis of the statistical analysis will be on between-group differences to establish if differences in outcome measures are apparent. This will be achieved using two-way repeated measures ANOVA analysis, followed by a series of post-hoc tests which are most likely to be t-tests.

All data will be tested for normality, and where normality does not occur the equivalent nonparametric tests will be used instead. A significance level of 0.05 will be used and a Bonferroni correction will be used when multiple t-tests are required. Qualitative data obtained from the various interviews will be collated and a suitable coding system established such that the responses can be categorised.

### Rigid AFO Design Criteria

Each rigid AFO used in the trial will be made to the following criteria:

1. The AFO must not position the ankle in a more dorsiflexed position than can be achieved with the knee fully extended (i.e. the gastrocnemius length). This means the AFO may hold the ankle in a plantar flexed position.

2. The AFO should give an initial shank-to-vertical angle of 0 degrees when placed on a flat surface without a shoe. A permanent wedge should be attached to achieve this if the AFO holds the ankle in a plantar flexed position.

3. 5 mm homopolymer polypropelene should be used.

4. Carbon fibre reinforcements should be used, with their leading edge placed at the midline of each malleolus.

5. There should be no noticeable deflection when the AFO is forced into plantarflexion or dorsiflexion. There should be no outward bulging at the malleoli when the AFO is forced into dorsiflexion.

6. Trimlines should be approximately 1 cm anterior to the midline of the malleioli. At the forefoot, the medial and lateral trimlines should be close to the metatarsal heads, to allow for control of supination/pronation. The sole plate should extend at least 5 mm beyond the toes.

7. Straps should be made of velco (or webbing backed with velcro). The top strap should be no more than 10-15 from the top of the AFO. The lower strap should apply a force in a posterior and inferior direction, at roughly 45 degrees to the vertical, to the dorsum of the foot.

### Ethical Approval

This study received ethical approval from NHS West of Scotland Research Ethics Committee 4 on 1^st ^April 2011 (Ref:11/AL/0166). NHS Greater Glasgow and Clyde R&D approval granted 19^th ^July 2011 (Ref: GN10OR216).

## Discussion

This protocol describes an early exploratory phase randomised controlled trial [29] which aims to assess the impact of using biomechanics visualisation in the context of AFO tuning with stroke patients.

**Figure 2:**
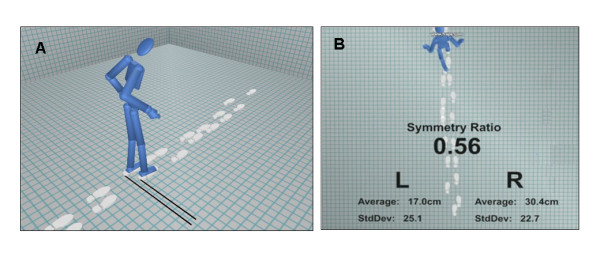
**Example visualisation screenshots showing data from one patient in two different ways**: a) the relationship between step length, gait symmetry and walking speed for one individual patient, and b) the same motion data from a different angle and with diagnostic numerical data for the clinician.

The visualisation software design team worked closely with bioengineers, physiotherapists and orthotists to gain an understanding of the AFO tuning process. This work involved informal research methods from conversations and observation of current working practices through to more formal focus group discussions. It is anticipated that an appropriate biomechanics visualisation software tool has been developed. The work suggested that only a small, very specific, subset of gait parameters is necessary for making clinical decisions and measuring progress for AFO tuning. The visualisation software tool will take the data collected with conventional 3D motion analysis equipment and display it in novel visual ways to benefit both clinicians and patients (see Figure 2). Only the smallest number of gait parameters necessary will initially be presented to clinicians to help them make quick and accurate clinical decisions based on objective numerical data. It is anticipated that this will go some way to addressing the aforementioned problems of interpreting gait analysis [16]. As well as potentially benefitting from any improved decision making from the clinicians, the patient may also benefit from having the advantages of their AFO explained to them and also having any progress they are making highlighted at the outcome and follow-up stages.

One particular strength of this study is that the authors have attempted to address the problem of previous studies failing to provide sufficient levels of detail when describing the participants recruited and/or the orthosis being investigated [30].

As this is a relatively small-scale study, it is limited slightly by only having two MDTs so there is a chance that the patient outcomes will be influenced by the skills knowledge and experience of the individual MDT members, and the resulting group dynamics. However, all MDT members will be practicing NHS professionals whose primary aim is to provide the best possible care for the patient, and as mentioned previously they will all be given some basic biomechanics refresher training before the trial commences.

While primarily focussed on gait related outcome measures, this study aims to develop a richer understanding of the effects of AFO use in stroke patients. Through the use of outcome measures such as the EQ-5D, Modified Rivermead Mobility Index, Modified Ashworth scale and various qualitative measures a clearer picture of what overall impact AFOs have on stroke patients lives should become apparent.

## Trial Status

The trial is ongoing.

## Abbreviations

SIGN: Scottish Intercollegiate Guidelines Network; AFO: Ankle-Foot Orthosis; NHS: National Health Service (UK); RCT: Randomised Controlled Trial; BPS: Best Practice Statement; GRF: Ground Reaction Force; 3D: Three dimensional; R&D: Research and Development; MDT: Multidisciplinary Team; ANOVA: Analysis of Variance.

## Competing interests

The authors declares they have no competing interests.

## Authors' contributions

BC has taken the lead role in developing the study protocol and in drafting and revising the manuscript. PR, BM and RB have all played significant roles in developing the study protocol, have helped revise the manuscript critically and have given final approval of the version to be published. All authors read and approved the final manuscript.
